# Risk factors for upper adjacent segment degeneration after multi-level posterior lumbar spinal fusion surgery

**DOI:** 10.1186/s13018-019-1126-9

**Published:** 2019-03-28

**Authors:** Zhaoxin Ma, Shilei Huang, Jianguang Sun, Feng Li, Jianhao Sun, Guofu Pi

**Affiliations:** Department of Orthopaedics, The First Affiliated Hospital of Zhengzhou University, No.1 Jianshe East Road, Zhengzhou, 450052 Henan China

**Keywords:** Adjacent segment degeneration, Multi-level, Posterior lumbar fusion, Disc degeneration, Pelvic incidence, Decompression, Risk factor

## Abstract

**Background:**

Posterior lumbar spinal fusion has been widely used in degenerative lumbar stenosis, but adjacent segment degeneration (ASD) was common. Researchers have found many risk factors for ASD after one or two levels of surgery, but few clinical studies focused on multi-level surgery. The purpose of this study was to clarify risk factors for upper ASD after multi-level posterior lumbar spinal fusion.

**Methods:**

A retrospective study was performed on the clinical data of 71 patients with degenerative lumbar stenosis who underwent multi-level (at least 3 levels) posterior lumbar spinal fusion from January 2013 to December 2016. Two groups were divided according to lamina and posterior ligamentous complex (PLC) maintenance of proximal fixed vertebrae in surgery. In the 22 patients of group A, the proximal fixed vertebral lamina and PLC were not resected, and in the 49 patients of group B, the proximal fixed vertebral lamina and PLC were resected completely. Age, sex, body mass index (BMI), number of fixed vertebrae and fused levels, spinopelvic parameters, coronal Cobb angle, and modified Pfirrmann grading system were measured for each patient. A Cox proportional hazards model was used to analyze risk factors for upper ASD.

**Results:**

No symptomatic ASD was found during the follow-up period. Patients who underwent proximal fixed vertebral lamina and PLC resection had a significantly higher percentage of radiographic ASD (*P* = 0.042). The Cox proportional hazards model showed that age, sex, BMI, preoperative lumbar lordosis, sacral slope, pelvic tilt, coronal Cobb angle, number of fixed vertebrae, and interbody fusion levels had no significant differences for radiographic ASD. But a preoperative modified Pfirrmann grade higher than 3, a high degree of preoperative pelvic incidence, and more decompressed levels had statistical significance (*P* = 0.024, 0.041, and 0.008, respectively).

**Conclusions:**

A preoperative modified Pfirrmann grade higher than 3, a high degree of preoperative pelvic incidence, and more decompressed levels might be risk factors for upper radiographic ASD after multi-level posterior lumbar spinal fusion surgery.

## Background

Degenerative lumbar stenosis (DLS) is a common disease in an elderly population, characterized by back pain, radicular or claudicant leg pain, and disability [1]. DLS usually has more than one canal stenosis level and goes with spinal scoliosis or imbalance. Surgical treatment such as posterior lumbar spinal fusion is an effective way to improve patients’ clinical symptoms and correct spinal function [2]. Posterolateral fusion (PLF) and posterior lumbar interbody fusion (PLIF) have been commonly performed procedures that have been associated with substantial functional improvements for DLS. However, after multi-level posterior lumbar spinal fusion, there may be new clinical troubles, such as adjacent segment degeneration (ASD) [3]. These troubles affect patients’ prognosis and quality of life.

ASD can be classified as symptomatic and radiographic [4]. Symptomatic ASD refers to new symptoms or signs of an adjacent segment which often needs a revision surgery procedure to address adjacent level pathology. Radiographic ASD, generally defined as radiographic changes of the adjacent segment, has no clinical symptoms [5]. There are many risk factors for ASD, such as age, sex, body mass index (BMI), surgery strategy, and sagittal alignment change [6]. But there are few clinical studies about the risk factors for ASD after multi-level posterior lumbar spinal fusion surgery.

In this retrospective study, to analyze the risk factors for upper ASD, we followed up 71 patients who underwent multi-level posterior lumbar spinal fusion surgery for about 3 years.

## Materials and methods

### General information

This retrospective study was approved by the Ethics Committee of The First Affiliated Hospital of Zhengzhou University. At preoperation, standing lumbar anteroposterior and lateral radiographs, computed tomographic (CT) scans, and magnetic resonance imaging (MRI) were taken to assess surgery segments for each patient. Inclusion criteria were the following: (1) multi-level (at least 3 levels) degenerative lumbar stenosis, which has coincidence imaging diagnosis and cardinal symptoms such as back and leg pain and intermittent claudication; (2) invalid after 3 to 6 months of conservative treatment; and (3) the surgical treatment were multi-level (at least three levels) posterolateral fusion (PLF) in combination with posterior lumbar interbody fusion (PLIF). Exclusion criteria were the following: (1) history of thoracolumbar trauma, thoracolumbar tuberculosis or infection, and cervical spondylosis cross-inducing pain in both lower limbs or walking dysfunction and (2) combined lower limb peripheral nerve injury. Seventy-one patients from January 2013 to December 2016 met the inclusion criteria.

### Groups

Patients were divided into two groups (A and B) according to the lamina and posterior ligamentous complex (PLC) maintenance of proximal fixed vertebrae in surgery. In group A, the proximal fixed vertebral lamina and PLC were not resected. In group B, the proximal fixed vertebral lamina and PLC were both resected completely.

### Surgical technique

All procedures were performed by the senior author and use general anesthesia, prone position, abdomen vacant, and posterior median approach. The laminae of the fixed segment were exposed such that the articular process and base of the transverse process were fully visualized. The correct pedicle screws were inserted into the fixed vertebrae. Decompression and fusion segments were determined based on preoperative symptoms, imaging findings, and signs in surgery. In group A, the proximal fixed vertebral lamina and PLC were not resected. In group B, both the proximal fixed vertebral lamina and PLC were resected. All patients underwent laminectomy by osteoma and rongeur. During the decompression, care must be taken to avoid facet joint injury. Lateral crypts and nerve root canals expanded clearly. After complete decompression, properly sized autologous bone particles and interbody fusion cages were inserted into the intervertebral space. After decortication using osteotome at the transverse process and lateral side of the facet joint of fixed vertebrae, the autologous bone harvested from the spinous process and laminae was placed into the gutter made here. C-arm perspective determined the ideal spinal balance.

### Postoperative treatment

Patients in both groups were treated with the same postoperative protocols. All patients had to exercise their lower limb function and turn over on the bed from the first day to 6 weeks after surgery. From 6 weeks to 3 months after the surgery, the patients were fitted with thorax lumbar sacrum orthosis (TLSO) and instructed to walk. Three months after the surgery, the patients were allowed to resume their normal activities without TLSO.

### Clinical and radiographic evaluation

Clinical outcomes were assessed by the Oswestry Disability Index (ODI) and the Japanese Orthopaedic Association (JOA) score, measured at preoperation and postoperation at 3 months, 6 months, and annually up to 3 years.

Standing lumbar anteroposterior and lateral radiographs were measured at preoperation and 3 months, 6 months, and annually up to 3 years after surgery. Lumbar lordosis (LL), pelvic incidence (PI), sacral slope (SS), pelvic tilt (PT), coronal Cobb angle (Cobb), disc height (DH), and slippage distance (SD) were measured on radiographs. The DH and SD were measured at the upper adjacent segment of fixed vertebrae. The DH was defined as the average value of the anterior, central, and posterior disc height. The SD was measured as reported [7]. Pre-existing upper disc degeneration on preoperative MRI was defined according to the modified Pfirrmann grading system [8]. All data was measured by two independent reviewers.

### Criteria for radiographic ASD

Radiographic ASD was defined as follows [9]: (1) DH reduction of more than 20% of the preoperative disc height, (2) SD more than 3 mm compared with the preoperative condition seen on lateral radiographs, and (3) osteophyte formation greater than 3 mm.

### Statistical analyses

Statistical analyses were performed with the use of SPSS 21.0 (SPSS, Inc.). Data were expressed as the mean ± standard deviation. The *t* test, Mann-Whitney *U* test, and Pearson chi-square test were used to analyze demographic differences, changes of disc height, slippage distance, JOA, ODI, and spinopelvic parameters. Risk factors for radiographic ASD were measured by the log-rank test. The Cox proportional hazards model was used to adjust for confounding variables, including age, sex, BMI, preoperative modified Pfirrmann grade, LL, PI, PT, SS, coronal Cobb angle (Cobb), number of fixed vertebrae, decompressed levels, and interbody fusion levels. A *P* value < 0.05 was considered statistically significant.

## Results

In this study, 71 patients (34 men, 37women) met the inclusion criteria in total, with a mean age of 62.2 years. There were 22 patients in group A (11 men, 11 women) and 49 patients in group B (23 men, 26 women). The age, sex, BMI, ODI, and JOA had no statistical differences between groups A and B. As for the preoperative modified Pfirrmann grade, the number ratio of ≤ 3 versus> 3 in group A was 16:6 and in group B was 19:30; there were statistically significant differences(*P* = 0.011, Pearson’s chi-square test). The mean number of fixed vertebrae, decompressed levels, and interbody fusion levels had statistical differences between groups A and B (Table 1). These numbers were detailed in Tables 2, 3 and 4.

**Table 1:** Demographic data

Demographic and pre-op data	Group A(*n* = 22)	Group B(*n* = 49)	*P* value
Age (years)	60.59 ± 9.03	62.96 ± 8.59	0.294
Sex(M:F)	11:11	23:26	0.811^#^
BMI (kg/m^2^)	25.28 ± 3.19	25.31 ± 3.78	0.975
Pre-op Pfi^◆^(no.)			0.011^#^
1	2	1	
2	10	10	
3	4	8	
4	3	18	
5	2	8	
6	1	4	
7	0	0	
8	0	0	
ODI			
Pre-op	52.93 ± 11.47	55.18 ± 10.62	0.426
Last follow-up	17.38 ± 8.38	15.66 ± 4.29	0.265
JOA			
Pre-op	3.5 ± 3.16	3.24 ± 3.36	0.764
Last follow-up	21.14 ± 3.53	21.06 ± 2.82	0.924
Fixed vertebrae (no.)	4.82 ± 1.00	4.22 ± 0.51	0.007*
Decompressed levels (no.)	2.36 ± 0.66	2.99 ± 0.72	0.001*
Interbody fusion (no.)	1.23 ± 0.92	2.00 ± 1.00	0.003*

^◆^Preoperative modified Pfirrmann grade for upper disc of proximal fixed vertebrae

^#^Pearson’s chi-square test

*Mann-Whitney *U* test

**Table 2:** Fixed vertebrae number comparison

Fixed vertebrae	Group A (*n* = 22)	Group B (*n* = 49)
T11–L3	2	0
T11–L4	1	0
T11–L5	1	0
T12–L5	3	1
L1–L5	2	4
L1–S1	1	1
L2–L5	9	26
L2–S1	1	2
L3–S1	2	15

**Table 3:** Decompressed levels number comparison

Decompressed levels	Group A (*n* = 22)	Group B (*n* = 49)
T12–L1	2	0
T12–L2	0	1
L1–L3	2	2
L1–L4	1	1
L2–L3	5	12
L2–L4	0	2
L2–L5	0	9
L3–L5	2	13
L3–S1	0	9
L4–L5	7	0
L4–S1	3	0

**Table 4:** Interbody fusion levels number comparison

Interbody fusion levels	Group A (*n* = 22)	Group B (*n* = 49)
Not fused	7	6
L2–4	0	2
L2–5	0	10
L3–5	9	13
L3–5 to S1	0	8
L4–5	3	5
L4–5 to S1	3	4
L5–S1	0	1

Twenty-nine patients (40.85%) presented with radiographic ASD at the last follow-up. Five cases (22.73%) came from group A, and 24 cases (48.98%) came from group B. The percentage of radiographic ASD was significantly higher in group B than in group A (*P* = 0.042, Pearson’s chi-square test). Clinical results had no statistically significant differences between groups A and B (Table 1). No symptomatic ASD was found during the follow-up.

As for the radiographic analysis at preoperation and last follow-up, LL, SS, PT, coronal Cobb angle, and ΔPILL had no statistically significant differences between groups A and B. But group B had a significantly higher degree of PI than group A (*P* = 0.042) at preoperation (Table 5) .

**Table 5:** Radiographic measurements(°)

	Group A (*n* = 22)	Group B (*n* = 49)	*P* value
Pre-op			
LL	34.09 ± 20.03	32.42 ± 14.90	0.695
PI	43.64 ± 10.10	49.29 ± 10.77	*0.042*
PT	18.44 ± 8.80	22.53 ± 9.44	0.092
SS	26.18 ± 9.28	26.96 ± 8.97	0.742
Cobb^a^	11.33 ± 5.24	9.97 ± 5.33	0.322
ΔPILL^b^	9.56 ± 17.30	17.07 ± 14.37	0.217
Last follow-up			
LL	35.86 ± 13.66	39.04 ± 10.61	0.240
PI	47.09 ± 14.53	49.47 ± 8.56	0.486
PT	15.20 ± 9.98	15.16 ± 6.13	0.985
SS	31.70 ± 10.67	34.75 ± 7.96	0.193
Cobb^a^	4.43 ± 2.23	5.30 ± 2.75	0.199
ΔPILL^b^	10.17 ± 13.73	9.73 ± 8.63	0.804

^a^Coronal Cobb’s angle

^b^ΔPILL = PI-LL

To analyze the risk factors for radiographic ASD, we performed the log-rank test and used the Cox proportional hazards model. The age, sex, BMI, preoperative LL, SS, PT, coronal Cobb angle, number of fixed vertebrae, and fused levels had no significant differences in the Cox proportional hazards model. But the upper disc had a preoperative modified Pfirrmann grade > 3, a high degree of PI at preoperation, and more decompressed levels which had statistically significant differences (Table 6).

**Table 6:** The Cox proportional hazards for the risk factor of ASD

Factor	Exp (B)^b^	95% CI^c^ for Exp (B)	*P* value
Pre-op Pfi^a^	2.914	1.149–7.392	0.024
Pre-op PI	1.040	1.002–1.080	0.041
Decompressed levels	2.080	1.208–3.580	0.008
Fixed vertebrae	0.634	0.303–1.326	0.226
Interbody fusion levels	0.976	0.616–1.548	0.918

^a^Preoperative modified Pfirrmann grade for upper disc of proximal fixed vertebrae

^b^Exp (B) = relative risk

^c^Confidence interval

**Fig. 1:**
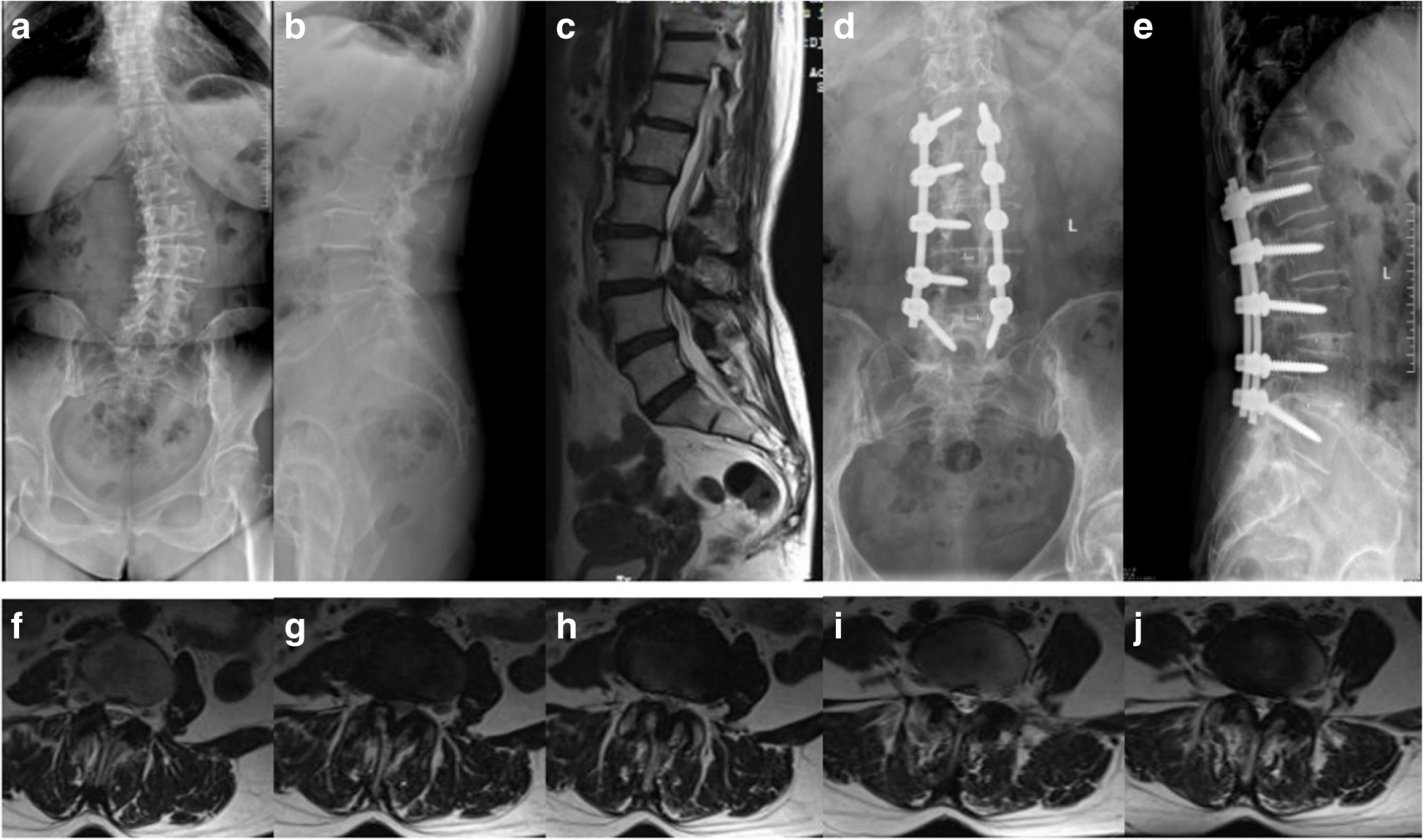
**a**, **b** Preoperative anteroposterior and lateral radiograph. **c** Preoperative sagittal MR image. **d**, **e** Anteroposterior and lateral radiograph 24 months after surgery showing no indication of radiographic ASD. **f**–**j** Preoperative coronal MR image

**Fig. 2:**
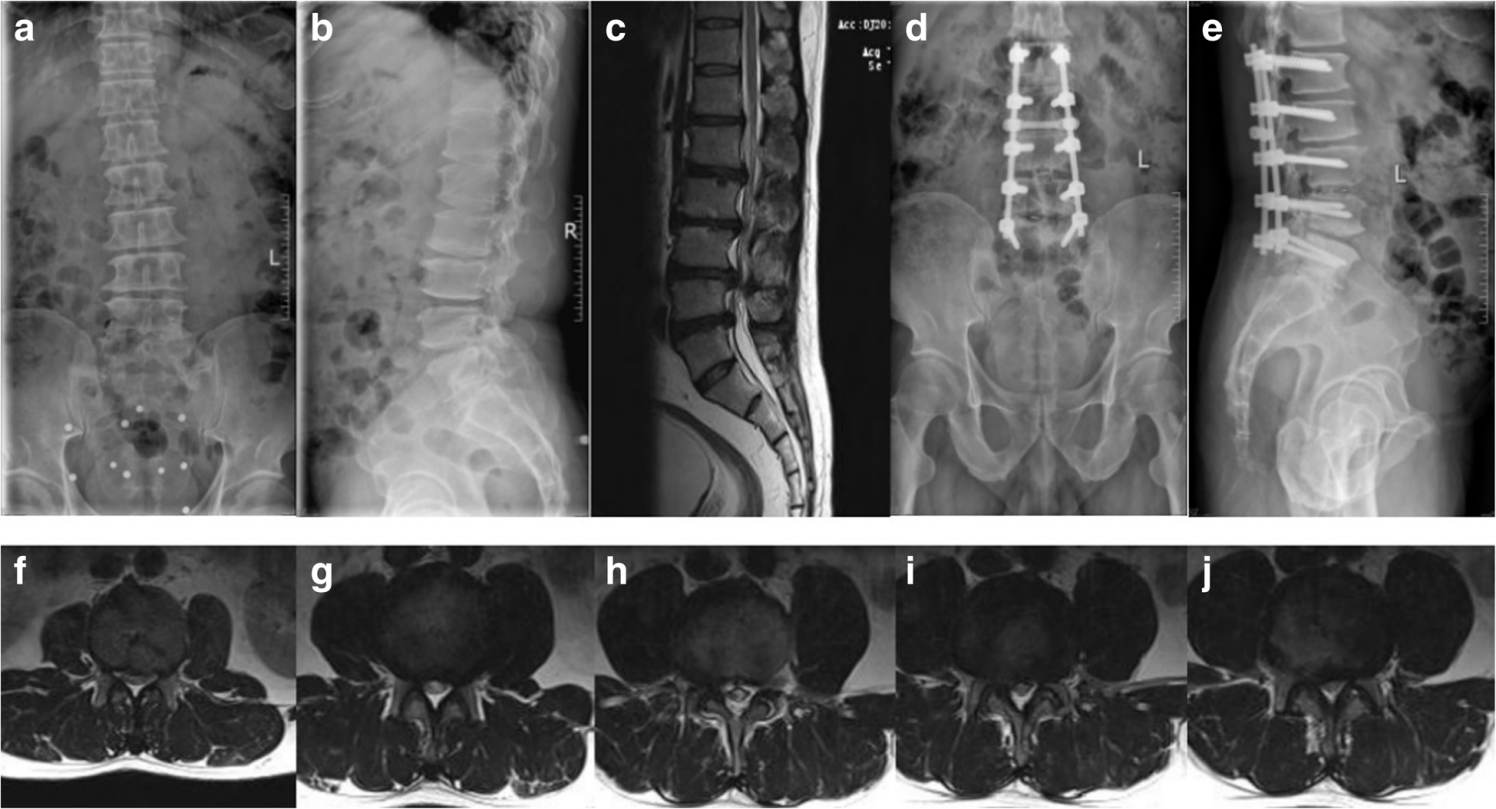
**a**, **b** Preoperative anteroposterior and lateral radiograph. **c** Preoperative sagittal MR image. **d**, **e** Anteroposterior and lateral radiograph 18 months after surgery showing radiographic ASD, T12/L1 disc height decreased from 7.50 to 6.0 mm. **f**–**j** Preoperative coronal MR image

There are two illustrative cases for groups A and B, respectively. Group A (Fig. 1): female, 58 years old, diagnosed with degenerative lumbar stenosis. L1–5 were fixed, L3–5 underwent laminectomy, L3–5 underwent interbody fusion, and L1–5 underwent posterolateral fusion. T12/L1 disc preoperative modified Pfirrmann grade was 4, and preoperative PI was 58°. No radiographic ASD of T12/L1 level was found at 24 months after surgery. Group B (Fig. 2): male, 60 years old, diagnosed with degenerative lumbar stenosis. L1–5 was fixed, L1–5 underwent laminectomy, L3–5 underwent interbody fusion, and L1–5 underwent posterolateral fusion. T12/L1 disc preoperative modified Pfirrmann grade was 5, and preoperative PI was 59.78°. Radiographic ASD of T12/L1 level was found because the T12/L1 disc height decreased from 7.50 to 6.0 mm at 18 months after surgery.

## Discussion

Degenerative lumbar stenosis (DLS) is a complex degenerative disease of the spine that often occurs in the elderly population. DLS may have more than one stenosis spinal levels or with spinal instability. Surgical treatment can restore spinal stability, relieve nerve root compression effectively, and improve symptoms and patients life quality [10]. The most ideal surgery treatment for degenerative lumbar stenosis or with scoliosis still remains controversial. But ASD which came from the posterior lumbar spinal fusion surgery always attracts the attention of spine surgeons. Many risk factors are involved in ASD, but the mechanism for its occurrence is still controversial. Moreover, there was no correlation between both the radiological development of ASD and its clinical outcome [11].

In this retrospective study, after multi-level posterior lumbar spinal fusion surgery, 29 patients (40.85%) presented with radiographic ASD, but we did not find symptomatic ASD for approximately 3 years. We observed that preoperative modified Pfirrmann grade > 3, a high degree of PI at preoperation, and more decompressed levels were the risk factors for radiographic ASD in this study.

Some researches have suggested that preoperative disc degeneration is related with radiographic ASD [9, 12]. Intrinsic disc degeneration causes the increase of disc stress at the adjacent segment [13, 14]. If this preoperative disc degenerative segment is not involved in fusion, it might aggravate the adjacent disc degeneration after fusion surgery [15]. In this study, we also find that pre-existing disc degeneration (modified Pfirrmann grade > 3) of the upper fixed segment is a risk factor for radiographic ASD(*P* = 0.024, 95% CI 1.149–7.392).

Sagittal alignment of the lumbar spine has an important and measurable impact on the pathogenesis of lumbar spine disorder and contributions to ASD [16]. Anatomic pelvic parameters also have an association with lumbar spinal degeneration, such as lumbar spinal stenosis and degenerative spondylolisthesis [17]. An appropriate sacral inclination is important for minimizing the incidence of ASD [18]. Di Martino et al. reported that patients with SS < 39° or PT > 21° were at higher risk for symptomatic ASD [19]. Moreover, a high degree of PI was a risk factor for developing early-onset radiographic ASD [20]. In addition, Rothenfluh et al. found that in degenerative disease of the lumbar spine, a high PI with diminished LL, especially ΔPILL ≥ 10°, seems to predispose to adjacent segment disease. If sagittal malalignment is maintained after lumbar fusion surgery, patients with such PI-LL mismatch exhibit a higher risk for undergoing revision surgery [21]. In this study, group B had a significantly higher preoperative PI than group A (49.29 ± 10.77° vs 43.64 ± 10.10°, *P* = 0.042), which contributes to a significant higher radiographic ASD ratio. The Cox proportional hazards model showed that a high PI is an independent risk factor for radiographic ASD ( *P* = 0.041, 95% CI 1.002–1.080). But there are no significant differences in SS, PT, or ΔPILL at preoperation and last follow-up. This reason might be that there is only radiographic ASD in this study but no symptomatic ASD.

The long-term RCT showed that compared with the effect of fusion with natural history, laminectomy and fusion accelerate degenerative changes at the adjacent level [22]. Patients who underwent fusion of three or four levels had a threefold increased risk of further surgery, compared with single-level fusions [23]. Biomechanical experiments found that the posterior column structure of the spine plays a key role in maintaining the stability of the spine [24]. Laminectomy of fusion segment head vertebra had been the reason for losing the balance in its flexion and axial rotation, which lead to the occurrence of ASD [25]. In this study, patients in group B, whose proximal fixed vertebral lamina and PLC were resected in surgery, had a significantly higher number of decompressed levels than group A. More decompressed levels mean that the proximal fixed vertebral posterior column structure was damaged more extensively. These also had contributions to a significantly higher radiographic ASD ratio in group B. But we did not find that the number of fixed vertebrae or interbody fusion levels have risk association with radiographic ASD. The reasons might be that (1) all patients in the two groups underwent multi-level fixation(≥ 4 fixed vertebrae) and (2) not all the proximal fixed vertebra underwent laminectomy and fusion together in group B. We just determined that more decompressed levels are a risk factor for radiographic ASD. Therefore, for patients with DLS requiring multi-level fixation and decompression, it is crucial to determine and handle the responsibility gap. In order to prevent ASD, surgeons must retain the posterior column structure such as lamina and PLC more entirely, determine the decompression range rationally, and protect the stability of the spine.

There are limitations in this study. More enrolled cases and more long-term follow-up period are needed for this study. Moreover, postoperative MR images and CT scans were not available for all patients, which might have affected our evaluation of ASD.

## Conclusion

Resection of proximal fixed vertebral lamina and PLC might accelerate the radiographic degeneration of their upper adjacent segment after multi-level posterior lumbar spinal fusion surgery (at least 3 levels). A preoperative modified Pfirrmann grade higher than 3, a high degree of PI at preoperation, and more decompressed levels might be the risk factors for upper radiographic ASD.

## Abbreviations

BMI: Body mass index
CI: Confidence intervals
DH: Disc height
LL: Lumbar lordosis
PI: Pelvic incidence
PLC: Posterior ligamentous complex
PT: Pelvic tilt
SD: Slippage distance
SS: Sacral slope
